# NSC666715 and Its Analogs Inhibit Strand-Displacement Activity of DNA Polymerase β and Potentiate Temozolomide-Induced DNA Damage, Senescence and Apoptosis in Colorectal Cancer Cells

**DOI:** 10.1371/journal.pone.0123808

**Published:** 2015-05-01

**Authors:** Aruna S. Jaiswal, Harekrushna Panda, Brian K. Law, Jay Sharma, Jitesh Jani, Robert Hromas, Satya Narayan

**Affiliations:** 1 Division of Hematology and Oncology, Department of Medicine, University of Florida, Gainesville, Florida, 32610, United States of America; 2 Department of Anatomy and Cell Biology, University of Florida, Gainesville, Florida, 32610, United States of America; 3 Department of Pharmacology and Experimental Therapeutics, University of Florida, Gainesville, Florida, 32610, United States of America; 4 Celprogen Inc., Torrance, California, 90503, United States of America; University of South Alabama Mitchell Cancer Institute, UNITED STATES

## Abstract

Recently approved chemotherapeutic agents to treat colorectal cancer (CRC) have made some impact; however, there is an urgent need for newer targeted agents and strategies to circumvent CRC growth and metastasis. CRC frequently exhibits natural resistance to chemotherapy and those who do respond initially later acquire drug resistance. A mechanism to potentially sensitize CRC cells is by blocking the DNA polymerase β (Pol-β) activity. Temozolomide (TMZ), an alkylating agent, and other DNA-interacting agents exert DNA damage primarily repaired by a Pol-β-directed base excision repair (BER) pathway. In previous studies, we used structure-based molecular docking of Pol-β and identified a potent small molecule inhibitor (NSC666715). In the present study, we have determined the mechanism by which NSC666715 and its analogs block Fen1-induced strand-displacement activity of Pol-β-directed LP-BER, cause apurinic/apyrimidinic (AP) site accumulation and induce S-phase cell cycle arrest. Induction of S-phase cell cycle arrest leads to senescence and apoptosis of CRC cells through the p53/p21 pathway. Our initial findings also show a 10-fold reduction of the IC_50_ of TMZ when combined with NSC666715. These results provide a guide for the development of a target-defined strategy for CRC chemotherapy that will be based on the mechanisms of action of NSC666715 and TMZ. This combination strategy can be used as a framework to further reduce the TMZ dosages and resistance in CRC patients.

## Introduction

Colorectal cancer (CRC) is the third most common cancer and the second leading cause of cancer death among American men and women (Cancer Facts and Figures 2014, American Cancer Society, Atlanta, GA). The current approach for discovering anti-tumor agents relies on semi-empirical screening procedures. However, the identification of agents through this method has proven to be ineffective in treating CRC due to an insufficient understanding of their pharmacology and their sum-total effect on the fate of cells in an *in vivo* environment, in the context of aberrant pathways, and in the tumor microenvironment [1–4].

It is well established that a compensatory DNA-repair capacity in tumor cells severely limits the efficacy of DNA-alkylating anti-cancer agents and, importantly, leads to recurrence of drug-resistant tumors [5–7]. The use of DNA-alkylating agents as chemotherapeutic drugs is based on their ability to trigger a cell death response [8] and their therapeutic efficacy is determined by the balance between DNA damage and repair. The DNA-alkylation damage-induced lesions are repaired by DNA polymerase β (Pol-β)-directed base excision repair (BER), O^6^-methylguanine DNA-methyltransferase (MGMT), and mismatch repair (MMR) pathways. Notably, the inhibitors that have been developed as anticancer drugs mainly target these three pathways [9, 10]. The active degradation product of DNA-alkylating prodrug-TMZ (NSC362856; 3,4-Dihydro-3-methyl-4-oxoimidazo[5,1-*d*]-1,2,3,5-tetrazine-8-carboxamide) is 5-(3-methyltriazen-1-yl)imidazole-4-carboxamide (MTIC) [11, 12], which methylates DNA at N^7^-methylguanine (N^7^meG), N^3^-methyladenine (N^3^meA), N^3^-methylguanine (N^3^meG) and O^6^-methylguanine (O^6^meG) in decreasing order of reactivity. BER is responsible for the repair of 70%, 5% and 9% of N^7^-MeG, N^3^-MeG, and N^3^-MeA lesions induced by the TMZ, respectively [13–16]; however, the potential utility of Pol-β as a target of the BER pathway blockade has not been explored.

In previous studies, we have shown that the small molecule NSC666715 [4-chloro-N-[5-(4-chloroanilino)-1H-1,2,4-triazol-3-yl]-5-methyl-2-sulfanylbenzenesulfonamide] mimics the interaction of adenomatous polyposis coli (APC) with Pol-β and flap endonuclease 1 (Fen1), blocks the Pol-β-directed BER pathway, and enhances the cytotoxicity of TMZ to CRCs [17]. TMZ produces strand breaks during BER-mediated repair of N^7^-MeG and N^3^-MeA adducts. The interruption of the BER pathway can contribute to the cytotoxicity of TMZ due to the accumulation of AP sites after the generation of DNA strand breaks [18]. TMZ-induced cell death has been reported to be mediated by several pathways depending upon the type of cancer cells and the concentration of the drug.

If the AP sites are not repaired, they accumulate and lead to single-strand DNA breaks (SSBs) that stall the DNA replication fork and form double-strand (and single-strand) DNA breaks during S phase. These unwound forks trigger apoptosis when they collapse to form one-sided double-strand DNA breaks (DSBs) [19]. Chemotherapy-induced DSBs are associated with senescence and apoptosis [20, 21]. In the present study, we examined how the blockade of the BER pathway by NSC666715 (and its analogs) might be involved in TMZ-induced AP site accumulation, and senescence and apoptosis in HCT116 CRC cells. Our central hypothesis is that the blockade of BER will induce significant accumulation of TMZ-mediated AP sites leading to senescence followed by the activation of caspase 3/PARP1 cleavage. This is predicted to result in CRC growth inhibition through apoptosis, caused by decreased levels of the anti-apoptotic protein, Bcl2, and increased levels of the pro-apoptotic protein, Bax [22, 23].

## Materials and Methods

### Maintenance of cells and treatment

HCT116 human colon cancer cell lines with wild-type *p53* gene (p53^+/+^) or with *p53* gene-knockout (p53^-/-^) or *p21* gene-knockout (p21^-/-^) were grown in McCoy's 5a medium supplemented with 10% fetal bovine serum (FBS; HyClone), 100 U/ml of penicillin, and 100 μg/ml of streptomycin. The HCT116 cell line was obtained from ATCC (Manassas, VA). This cell line was utilized because it is resistant to alkylating agents due to MMR deficiency. The HCT116(p21^-/-^) and HCT116(p53^-/-^) cell lines were provided by Dr. Bert Vogelstein (Johns Hopkins University) [24, 25].

### Oligonucleotides and Chemicals

**Fig 1:**
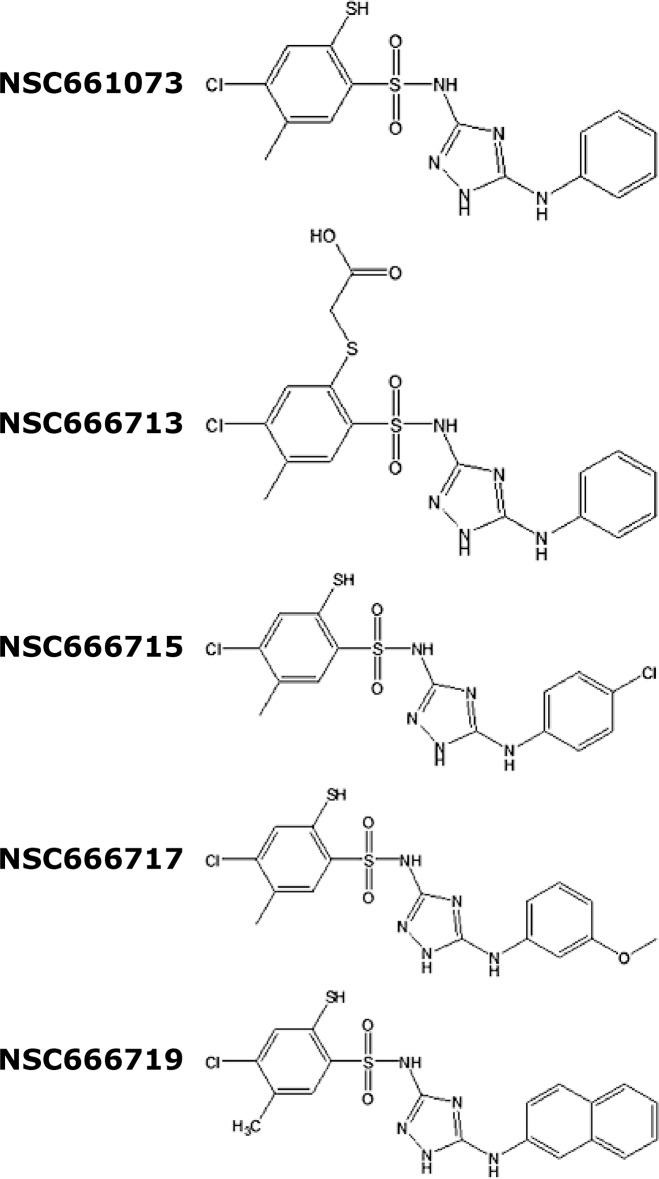
Chemical structure of the small molecule inhibitors. The chemical structures of the NSC666715 and its analogs NSC661073, NSC666713, NSC666717 and NSC666719 have been drawn using the ChemDraw software.

Oligonucleotides for the long-patch (LP)-BER assay were purchased from Sigma-Genosys (Woodlands, TX). T4-polynucleotide kinase (PNK) was purchased from New England Biolabs (Ipswich, MA) and radionuclide [γ-^32^P]ATP was purchased from Perkin Elmer, Inc. (Boston, MA). Small molecule inhibitors (SMIs) NSC666715 and its analogs NSC661073 [N-(5-anilino-1H-1,2,4-triazol-3-yl)-4-chloro-5-methyl-2-sulfanylbenzenesulfonamide], NSC666713 [2-[2-[(5-anilino-1H-1,2,4-triazol-3-yl)sulfamoyl]-5-chloro-4-methylphenyl]sulfanylacetic acid], NSC666717 [4-chloro-N-[5-(3-methoxyanilino)-1H-1,2,4-triazol-3-yl]-5-methyl-2-sulfanylbenzenesulfonamide], and NSC666719 [4-chloro-5-methyl-N-[5-(naphthalen-2-ylamino)-1H-1,2,4-triazol-3-yl]-2-sulfanylbenzenesulfonamide], and TMZ were obtained from the Developmental Therapeutics Program of the National Cancer Institute of the National Institutes of Health (DTP, NCI-NIH). The chemical structure of these SMIs is shown in Fig 1.

### Synthesis and Labeling of DNA Substrates

To examine the effect of SMIs on Pol-β-directed strand-displacement and LP-BER activities, a 63-mer oligonucleotide was synthesized as described earlier [26]. The nucleotide sequence of this oligonucleotide contains an AP site analog known as F (3-hydroxy-2-hydroxymethyltetrahydrofuran), which is positioned at 24-nt and referred as F-DNA (5’-CTAGATGCCTGCAGCTGATGCGC**F**GTACGGATCCACGTGTACGGTACCGAGGGCGGGTCGACA-3’). F-DNA was gel purified and labeled with [γ-^32^P]ATP at the 5′-end using T-4 polynucleotide kinase and annealed to a complementary oligonucleotide strand.

### *In vitro* strand-displacement synthesis and LP-BER Assay

The Pol-β–directed strand-displacement assay reaction mixture was assembled in a 30 μl volume with 30 mM Hepes, pH 7.5, 30 mM KCl, 8.0 mM MgCl_2_, 1.0 mM DTT, 100 μg/ml BSA, 0.01% (v/v) Nonidet P-40, 2.5 nM of ^32^P-labeled 63-mer F-DNA substrate, 2 nM of AP endonuclease 1 (APE1), 5 nM of Pol-β and 0–125 μM of SMIs. The LP-BER reaction was reconstituted using purified proteins in a final reaction volume of 30 μl containing 30 mm Hepes, pH 7.5, 30 mm KCl, 8 mm MgCl_2_, 1 mm dithiothreitol, 100 μg/ml bovine serum albumin, 0.01% Nonidet P-40, 0.5 mm ATP, and 10 μm each of dATP, dCTP, dGTP and dTTP. The reaction mixture was assembled on ice by the addition of 0.5 nm APE1, 2.5 nm Pol-β, 10 nm flap endonuclease 1 (Fen1), and 100 nm DNA ligase I and then incubated for 5 min. The reactions were initiated by the addition of 2.5 ng of ^32^P-labeled 63-mer F-DNA followed by incubation at 37°C for 45 min. The reaction was terminated by the addition of stop buffer (0.4% (w/v) SDS, 5 mm EDTA, 1 μg of proteinase K) and incubated at 37°C for an additional 30 min [17, 26–29]. After incubation at 37°C, the DNA was recovered by phenol/chloroform extraction and ethanol precipitation. The recovered DNA was washed with cold 70% ethanol and suspended in sample loading dye. The reaction products were separated on a 15% acrylamide and 7 M urea gel. The radioactive signals were visualized by autoradiography.

### Western blot analysis

For Western blot analysis, single-cell suspensions of HCT116 cells were plated (0.5 x 10^6^ cells per 60 mm dish) in triplicate. After 24 h, once the cells were attached to the plates, they were treated with small molecule inhibitor(s) alone or in combination with TMZ for 48 h. Changes in protein levels subsequent to the treatment of SMI’s were determined by Western blot analysis using whole-cell extracts. The antibodies used to detect the levels of p53, p21, Bcl2, Bax, Poly [ADP-ribose] polymerase 1 (PARP-1), cleaved PARP1, cleaved caspase 3, caspase 3, apoptosis inducing factor (AIF) and GAPDH were obtained from Cell Signaling Technologies (Danvers, MA).

### Estimation of AP sites in genomic DNA

For the estimation of the number of AP sites, a single-cell suspension of HCT116 cells was plated (0.5 x 10^6^ cells per well) in triplicate in six-well plates. Once the cells were attached to the plates, they were pretreated for 2 h with 25 μM of NSC666715, NSC666717 and NSC666719 followed by 500 μM of TMZ treatment for an additional 48 h. Cells were harvested and the AP sites were determined using the procedure described in previous studies [30, 31]. Genomic DNA from the treated and untreated groups was isolated using the GenElute Mammalian Genomic DNA isolation Kit (Sigma-Aldrich, St. Louis, MO). Five to 10 μg of the genomic DNA in 150 μl of 1x PBS was incubated with 1 mM aldehyde reactive probe (ARP) (Cayman Chemicals, Ann Arbor, MI) at 37°C for 10 min, then ethanol precipitated and finally dissolved in 1x TE buffer (10 mM Tris-HCl, 1 mM EDTA, pH 7.2) and quantified.

The ARP reacts with the AP site-containing genomic DNA and forms a complex, which can be quantitatively detected using chemiluminescent detection. Briefly, one μg of the ARP-treated heat-denatured DNA was slot-blotted onto a positively charged nylon membrane (Amersham Corp., Piscataway, NJ). The nylon membrane was soaked with 5x SSC (0.75 M NaC1, 0.075 M trisodium citrate) at 37°C for 15 min, briefly air-dried and baked in a vacuum oven at 80°C for 1–2 h. The membrane was preincubated with 10 ml of Tris-NaC1 buffer containing BSA (20 mM Tris-HCI (pH 7.5), 0.1 M NaC1, 1 mM EDTA, 0.5% casein, 0.25% BSA, and 0.1% Tween 20) at room temperature for 1 h. The membrane was then incubated in the same solution containing streptavidin-conjugated horseradish peroxidase (BioGenex, San Ramon, CA) at room temperature for 30–45 min. The membrane was rinsed three times for 10 min each with washing buffer (0.26 M NaC1, 1 mM EDTA, 20 mM Tris-HC1, and 0.1% Tween 20, pH 7.5), and the horseradish peroxidase enzymatic activity on the membrane was visualized using ECL reagent (Amersham Corp., Piscataway, NJ). The membrane was then exposed to X-ray film (Kodak XAR 5x; Kodak) for 5–10 sec. The developed film was analyzed for quantitation of the AP sites using the ImageJ program (Rasband, W.S., ImageJ, U. S. National Institutes of Health, Bethesda, Maryland, USA, http://imagej.nih.gov/ij/, 1997–2014). All experiments were performed in triplicate.

### Senescence associated β-galactosidase activity assay (SA-βgal Assay)

Senescence associated-β-gal activity was measured as described previously [32, 33] with minor modifications [34]. HCT116 cells were pretreated for 2 h with SMIs followed by TMZ treatment for an additional 48 h. Cells in sub-confluent cultures were washed with ice-cold phosphate-buffered saline (PBS), fixed in 4% (v/v) paraformaldehyde in PBS for 10 min at room temperature, and washed again three times with PBS. Cells were incubated with freshly made staining solution containing 1 mg/ml 5-bromo-4-chloro-3-indolyl β-D-galactoside (X-gal), 40 mM citric acid-sodium phosphate (pH 6.0), 5 mM potassium ferricyanide, 5 mM potassium ferrocyanide, 150 mM NaCl, and 2 mM MgCl_2_ for 24 h at 37°C. The blue-stained cells were observed under the microscope (Leica DMI 4000B). The number of senescence-associate β-gal positive cells was observed and analyzed under a microscope (Leica DMI 4000B), and the number of SA-β-gal-positive cells (blue colored cells) was counted in a total captured area of the image. We arbitrarily set the visual limit to exclude the false positive cells as the background for the SA-β-gal-positive cells in a defined area on the captured image. Results are expressed as percent of SA-β-gal-positive cells to total cells [35].

### Fluorescence activated cell sorting (FACS)

For the determination of the cell cycle progression profile, we plated HCT116 cells in 60 mm tissue culture dishes and grew them until they reached 60% confluence. HCT116 cells were pretreated for 2 h with NSC666715 (50 μM) and PFTα (10–30 μM) followed by TMZ (500 μM) for an additional 48 h as indicated in the Table 1 legend. Cells were harvested, washed once with ice-cold PBS, and processed for FACS analysis as described previously [36]. The cellular DNA content was analyzed using a Becton-Dickinson FACScan flow cytometer (San Jose, CA). At least 10,000 cells per sample were considered in the gated regions used for calculations. The ranges for the G_0_/G_1,_ S, G_2_/M and sub-G_1_ phase (apoptotic) cells were established based upon their corresponding DNA contents of the histograms. Results were analyzed and expressed as percentages of the total gated cells using the ModfitLT 3.1 program (Verity Software House, Topsham, NE).

**Table 1:** Effect of PFTα on the cell cycle phases of HCT116 cells treated with TMZ and NSC666715 either alone or in combination.

	% change			
Treatment	G_0_/G_1_	S	G_2_/M	sub-G_1_ (Apoptosis)
Control	83 ± 4.4	10 ± 2.8	7 ± 1.9	3 ± 1.3
500 μM TMZ	54 ± 1.6^§^	33 ± 1.2^§^	13 ± 1.3	6 ± 1.3
50 μM NSC666715	83 ± 1.4	11 ± 2.1	6 ± 0.7	2 ± 0.2
10 μM PFTα	61 ± 5.9^§^	29 ± 3.6^§^	10 ± 2.3	5 ± 1.1
20 μM PFTα	73 ±. 07	20 ± 1.1^§^	7 ± 0.6	5 ± 2.3
30 μM PFTα	79 ± 2.7	13 ± 2.1	7 ± 0.8	3 ± 0.4
10 μM PFTα + 50 μM NSC666715	73 ± 1.3	19 ± 1.3^§^	8 ± 0.7	6 ± 2.1
20 μM PFTα + 50 μM NSC666715	68 ± 5.5	21 ± 2.8	11 ± 2.9	7 ± 1.8
10 μM PFTα + 500 μM TMZ	67 ± 1.7^§^	24 ± 1.0^§^	9 ± 0.9	8 ± 3.5
20 μM PFTα + 500 μM TMZ	69 ± 3.1	21 ± 1.8^§^	10 ± 1.5	8 ± 3.3
30 μM PFTα + 500 μM TMZ	75 ± 5.8	18 ± 2.1	8 ± 3.8	7 ± 2.8
10 μM PFTα + 500 μM TMZ +50 μM NSC666715	69 ± 1.0^§^	21 ± 0.2^§^	10 ± 0.8	8 ± 1.9
20 μM PFTα + 500 μM TMZ +50 μM NSC666715	69 ± 5.5	20 ± 4.5	11 ± 0.5	10 ± 2.3
30 μM PFTα + 500 μM TMZ +50 μM NSC666715	69 ± 2.1^§^	21 ± 3.5	11 ± 1.5	9 ± 1.6

Cells were treated with different concentrations of drugs as indicated for 48 h. Cell cycle status and apoptosis were examined by FACS analysis. The percent distribution of cells in the G_0_/G_1_, S, G_2_/M and sub-G_1_ (apoptotic) phases of the cell cycle were established on the basis of the corresponding DNA content of the histograms. At least 10,000 cells per sample were considered in the gated regions for calculations. Data are presented as mean ± SE of three different estimations.

^§^ = Significantly different than the control (p <0.05).

### Statistical analysis

All experiments were repeated at least three times and results were expressed as mean ± SE. One Way analysis of variance (ANOVA) was calculated with SigmaPlot 9 (Systat Software, San Jose, CA). A one-tailed t-test was used to compare any significant difference between control and treated groups. The criterion for statistical significance was p<0.05. For western blotting results, the band intensities were measured by using the ImageJ and normalized with GAPDH.

## Results

### Pol-β inhibitor NSC666715 and its analogs inhibit LP-BER in an *in vitro* reconstituted system

**Fig 2:**
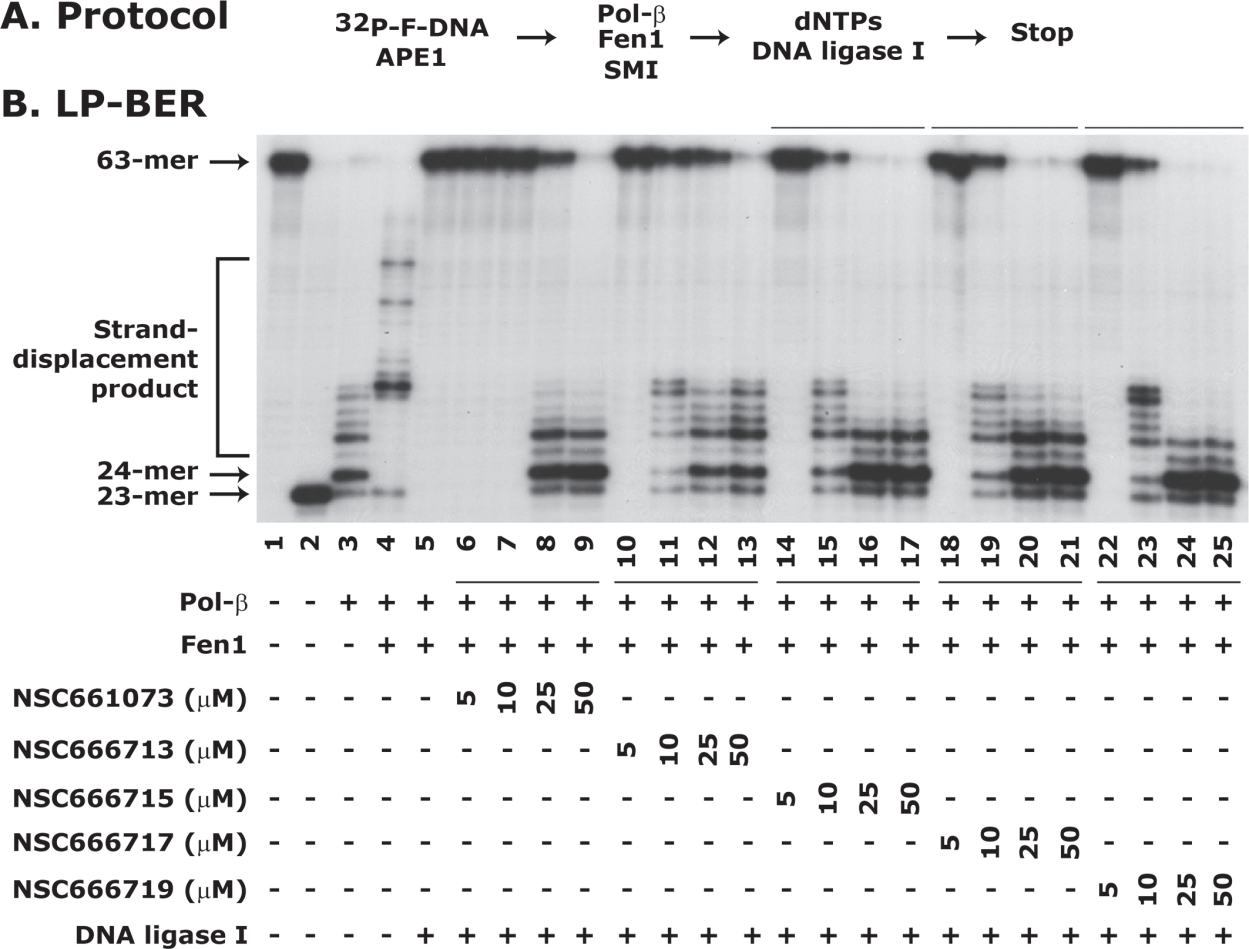
NSC666715 and its analogs block Pol-β-directed LP-BER activity. The LP-BER was determined using an *in vitro* reconstituted assay system. ***Panel A*** shows the experimental protocol. The assay details are given in Materials and Methods. ***Panel B*** shows an autoradiogram of LP-BER, which is representative of three different experiments. Lane 1 shows ^32^P-labeled 63-mer F-DNA and Lane 2 shows the 23-mer product after APE1 incision. Lane 3 shows Pol-β-mediated strand-displacement synthesis. Lane 4 shows Pol-β-mediated strand-displacement synthesis stimulated by Fen1 (Lane 4). Lane 5 shows the complete repair of the 63-mer F-DNA through the LP-BER pathway in the presence of APE1, Pol-β, Fen1 and DNA ligase I. Lanes 6–9, 10–13, 14–17, 18–21 and 22–25 show the effect of different concentrations of the Pol-β inhibitors NSC 661073, 666713, 666715, 666717 and 666719 on LP-BER activity.

In the present study, we tested several analogs of NSC666715, such as NSC661073, NSC666713, NSC666717, and NSC666719 for their ability to block LP-BER. The representative LP-BER results are shown in Fig 2. The appearance of the 23-mer incision product in Lane 2 indicates the functional activity of the APE1 protein. Pol-β-mediated 1-nt incorporated 24-mer product in Lane 3 and strand-displacement products in Lane 4. The stimulation of strand-displacement synthesis of Pol-β by Fen1 is an established feature of the Fen1-mediated LP-BER [28, 29, 37, 38]. In these experiments, we showed that the SMIs reduced Fen1-mediated strand-displacement activity of Pol-β (Fig 2, compare lane 5 with 6–9, 10–13, 14–17, 18–21, and 22–25, respectively), a consequence of blocked LP-BER (Fig 2, compare the 63-mer repaired product of lane 4 with 6–9, 10–13, 14–17, 18–21, and 22–25, respectively). The SMIs further showed the blockade of LP-BER at 50 μM; however, the maximum comparable blockade seen at lower concentrations was by NSC666715 and its two analogs NSC666717 and NSC666719 (Fig 2, compare lane 5 with 14–17, 18–21 and 22–25, respectively).

### Pol-β strand-displacement inhibitors increase the burden of AP sites in CRC cells after TMZ treatment as a consequence of cellular toxicity

**Fig 3:**
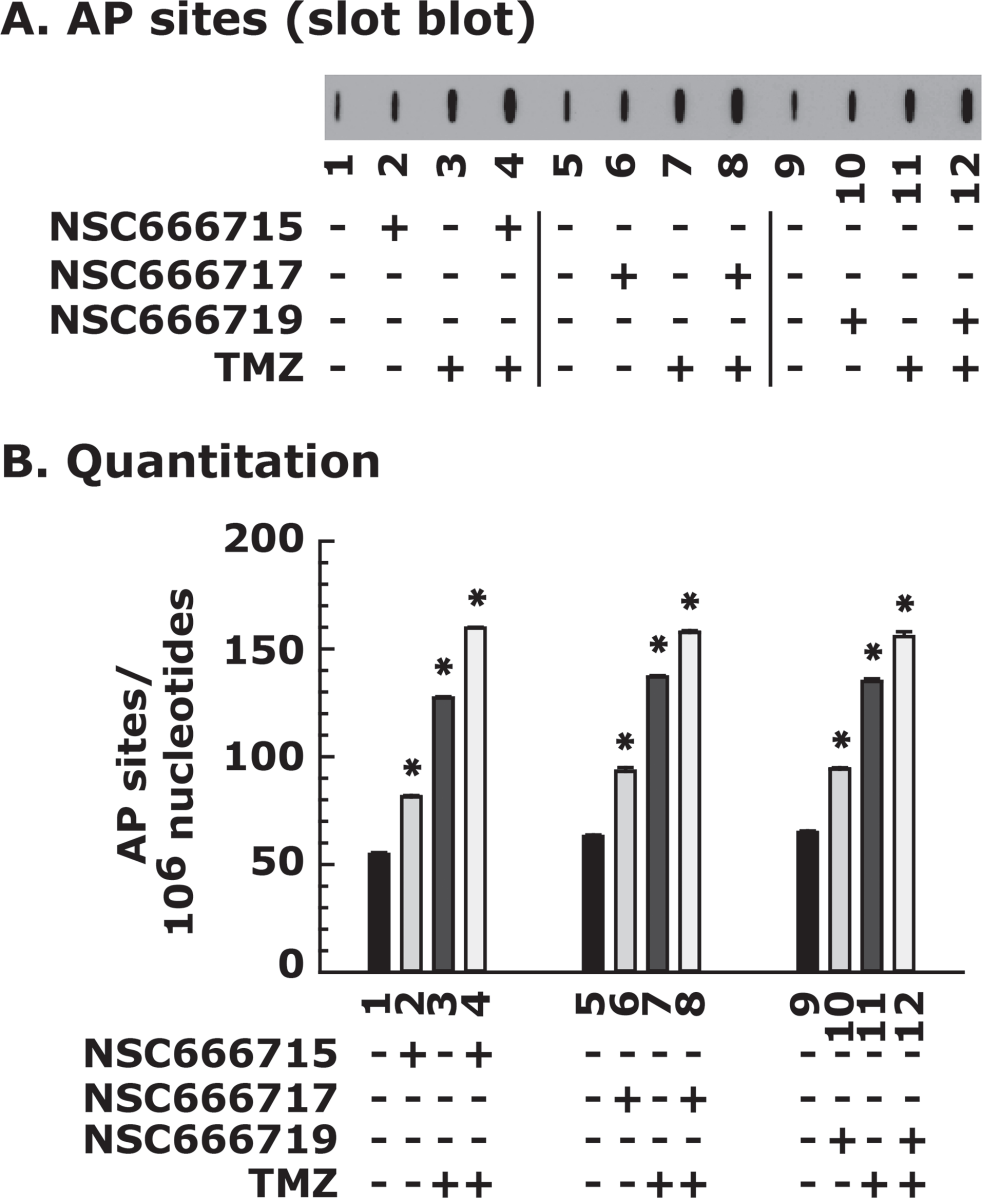
NSC666715 and its analogs cause the accumulation of TMZ-induced AP sites in the HCT116 cell line. Cells were pretreated for 2 h with 25 μM of the small molecule inhibitors NSC666715, NSC666717 and NSC666719, followed by treatment with 500 μM of TMZ for an additional 48 h. Genomic DNA was isolated and processed for AP site determination as described in Materials and Methods. ***Panel A*** shows the slot blot analysis of AP sites in HCT116 cells treated with 25 μM of NSC666715, NSC666717 and NSC666719 and 500 μM TMZ. ***Panel B*** shows the quantitative analysis of AP sites. Results are presented as mean ± SE of three different determinations. * = Significantly different than the untreated control group (P<0.05).

In these experiments, we determined the extent of DNA damage or the generation of AP sites after TMZ treatment in the presence or absence of SMIs in the HCT116 cell line. The tested SMIs (NSC666715, NSC666717 and NSC666719) showed an increase in AP sites (Fig 3, compare lane 1 with 2), and the burden of AP sites was further increased by combination treatment with TMZ (Fig 3, compare lane 1, with 3 and 4, respectively). Since the SMIs block the Pol-β pathway and do not interfere with the MMR pathway, as expected there was no significant difference on the level of AP sites in both MMR-deficient and MMR-proficient HCT116 cell lines after TMZ treatment alone or in combination with SMIs (data not shown). These results suggest that the SMIs NSC666715, NSC666717, and NSC666719 are specific for Pol-β-directed blockade of the BER pathway, and are therefore involved in TMZ-induced accumulation of AP sites.

### TMZ induces p21 levels via the p53 pathway

**Fig 4:**
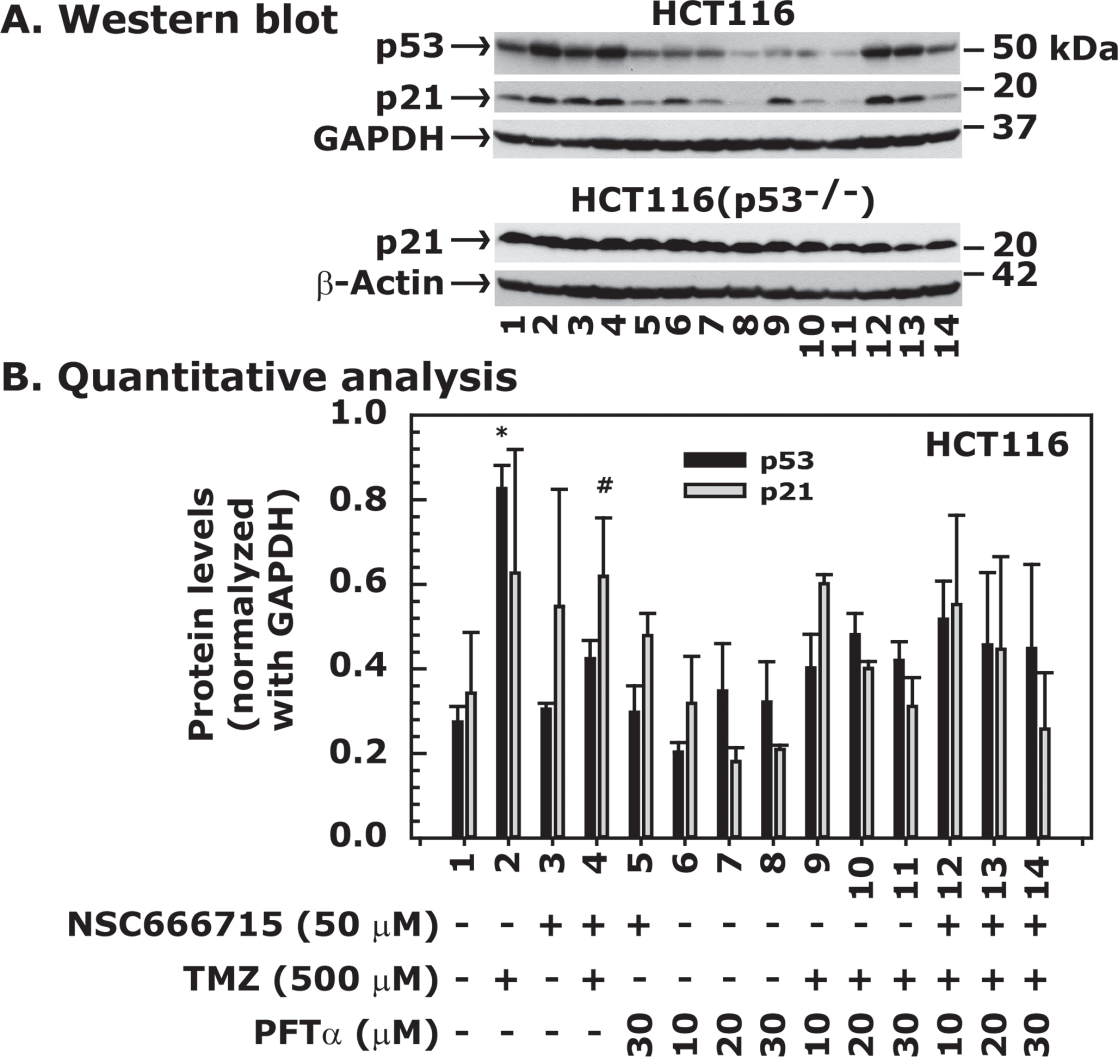
PFTα inhibits p53-mediated expression of p21 in HCT116 cells treated with TMZ and NSC666715. The HCT116 cells were pretreated with different concentrations of PFTα and 50 μM NSC666715 for 2 h and then with 500 μM TMZ alone or in combination for an additional 48 h. Cells were harvested, and cellular lysates were prepared and processed for Western blot analysis. **Panel A** shows immunoblots of the p53, p21, GAPDH and β-actin proteins. **Panel B** is the quantitative representation of p53 and p21 protein levels after normalization with GAPDH. Lane 1 shows p53 and p21 protein levels from the untreated control group and in Lane 2 and 3 after treatment with 50 and 500 μM NSC666715 and TMZ, respectively. Results in Lane 4 are from combination treatment with 50 μM NSC666715 and 500 μM TMZ. Results in Lane 5 are from the combination treatment with 50 μM NSC666715 and 30 μM PTFα. Results in Lanes 6–8 show p53 and p21 levels after treatment with 10, 20 and 30 μM PFTα, respectively. Results in Lanes 9–11 and 12–14 are from combination treatments with either 500 μM TMZ and 10, 20 and 30 μM PFT, or with 50 μM NSC666715, 500 μM TMZ and 10, 20 and 30 μM PFT, respectively. The data are presented as mean ± SE of three different experiments. * and ^#^ = Significantly different than the untreated control (P<0.05).

To determine whether TMZ activates the p53/p21 pathway and whether NSC666715 shows any effect on this pathway, we treated HCT116 cells with TMZ alone or in combination with NSC666715. The results showed a significant increase in both p53 and p21 levels after TMZ treatment alone (Fig 4A and 4B, compare lane 1 with 2). Treatment with NSC666715 alone had no effect on p53 levels, but p21 levels increased (Fig 4A and 4B, compare lane 1 with 3). This suggests that NSC666715 may require very little p53 activity for p21 activation, or induce p21 through a p53-independent mechanism. The combination of TMZ and NSC666715 increased p53 and p21 levels, but to a similar extent as their individual treatments (Fig 4A and 4B, compare lanes 1–4).

To further understand whether the increase in p21 levels after TMZ and NSC666715 treatment was p53-dependent, we determined p53 and p21 levels in HCT116 cells (containing wild-type p53 and p21) and HCT116(p53^-/-^) cells (p53-knockout and wild-type p21) with TMZ alone or in combination with NSC666715. The results showed a significant increase in both p53 and p21 levels after TMZ treatment alone (Fig 4A and 4B, compare lane 1 with 2) in HCT116 cells. To examine whether the TMZ-mediated increase in the p21 level was caused by p53, we treated HCT116(p53^-/-^) cells with TMZ and determined p21 levels. The results showed no increase in p21 in HCT116 (p53^-/-^) cells after TMZ treatment, suggesting that functional p53 is required for p21 induction (Fig 4A). To further verify this, we pre-treated HCT116 cells with pifithrin-α (PFTα), a p53 inhibitor that down-regulates *p21* gene expression [39], followed by the treatment with TMZ. The results showed that treatment with PFTα alone in HCT116 cells stabilized p53 and decreased p21 levels while p21 in HCT116(p53^-/-^) cells remained unaffected (Fig 4A, compare lanes 6–8) indicating that p53 is involved in p21 regulation (Fig 4A and 4B, compare lanes 6–8). PFTα also decreased p21 levels in a dose-dependent manner after treatment with TMZ alone or in combination with NSC666715 (Fig 4A and 4B, compare lanes 9–11 and 12–14, respectively). PFTα and NSC666715 treated cells showed some accumulation of p53 and p21 proteins (Fig 4A and 4B, lane 5), suggesting that NSC666715 may also require the p53/p21 pathway for its activity.

### TMZ-treated HCT116 cells that are arrested in the S-phase of the cell cycle are released by PFTα treatment

We addressed the role of p53 in cell cycle arrest following NSC666715 and TMZ treatment. We pre-treated HCT116 cells with different concentrations of PFT-α followed by TMZ treatment for FACS analysis. The FACS analysis results showed a significant S-phase arrest of cells after TMZ treatment, which was reduced in the presence of PFTα in a concentration-dependent manner (Table 1). While there was no effect of NSC666715 treatment alone, PFTα treatment caused a significant S-phase arrest at lower concentrations. However, at higher PFTα concentrations, the cells were released from S-phase and accumulated in the G_1_-phase. PFTα treatment reduced the S-phase arrest of HCT116 cells after treatment with TMZ or in combination with NSC666715. PFTα and NSC666715 treatment together did not have any effect on the S-phase arrest. The accumulation of cells in the G_2_/M phase prior to apoptosis began 48 h after treatment with either TMZ alone or in the presence of NSC666715, but the effect was not significant. These results suggest that TMZ induces an S-phase cell cycle arrest involving the p53 signaling pathway, which can be abrogated by PFTα. However, we recognize that PFTα effects may result from both p53-dependent and-independent mechanisms.

### NSC666715 enhances TMZ-induced senescence in HCT116 cells

**Fig 5:**
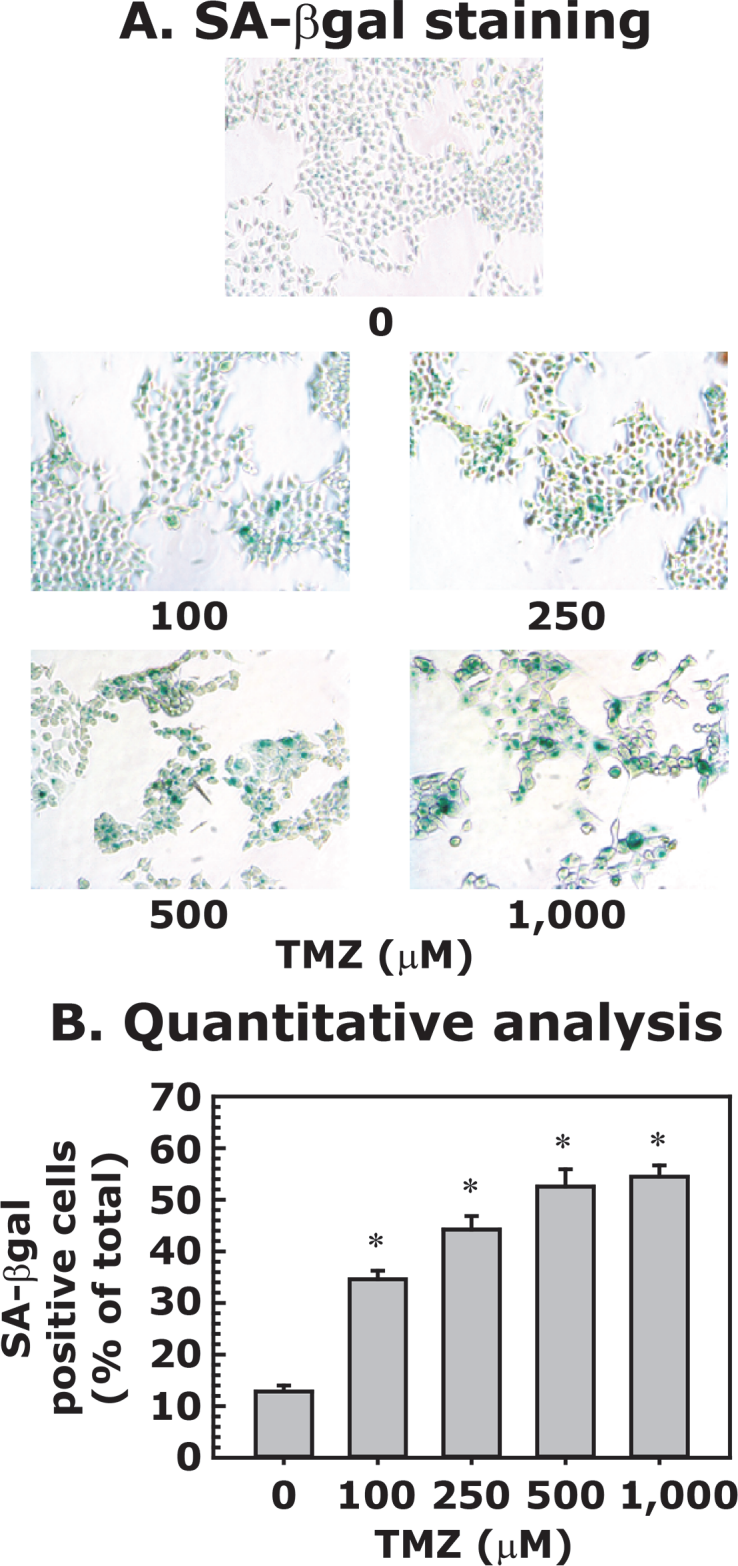
Effect of TMZ on senescence in HCT116 cells. HCT116 cells were treated with different concentrations of TMZ for 48 h and then processed for SA-βgal staining. **Panel A** shows SA-βgal staining. **Panel B** represents the quantitative analysis of SA-βgal staining. Data are presented as mean ± SE of four different estimations. * = Significantly different than the untreated control (p<0.05).

**Fig 6:**
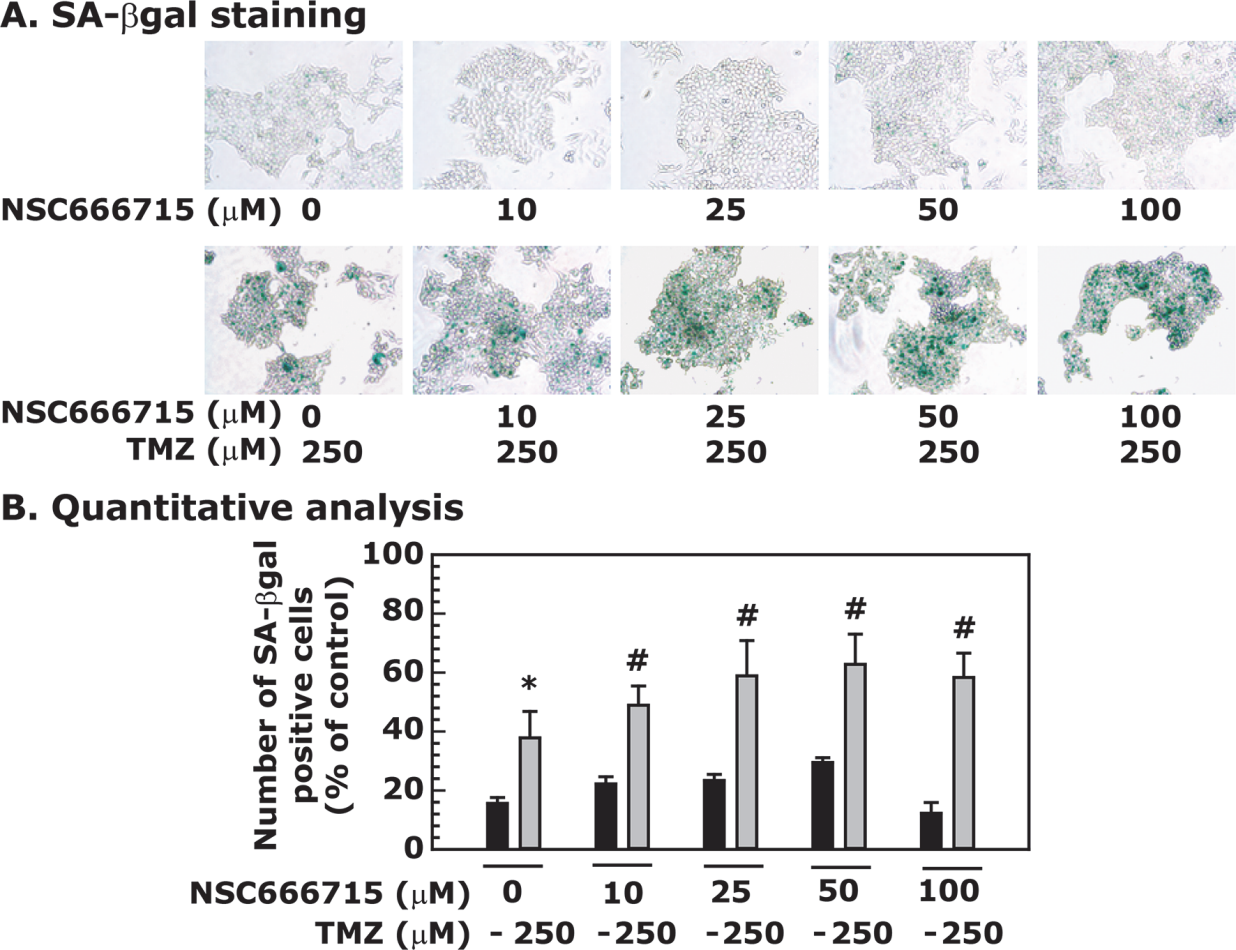
NSC666715 enhances TMZ-induced senescence in HCT116 cells. The HCT116 cells were pretreated with different concentrations of NSC666715 for 2 h followed by treatment with 250 μM of TMZ for an additional 48 h. After treatment, the cells were processed for SA-βgal staining. **Panel A** shows SA-βgal staining. **Panel B** represents the quantitative analysis of SA-β gal staining. Data are presented as mean ± SE of four different estimations. * and ^#^ = Significantly different than the untreated control or the 10, 25, 50 and 100 μM NSC666715 treated groups, respectively, (p<0.05).

First, we determined whether TMZ can induce senescence in HCT116 cells. Results showed an increase in SA-βgal staining; an indicator of senescence, in TMZ-treated HCT116 cells (Fig 5A and 5B). NSC666715 alone did not significantly induce senescence in colon cancer cells. However, NSC666715 in combination with TMZ triggered and increased frequency of senescence associated β-gal positive cells in a dose-dependent manner and continued to show statistically significant increase in senescence. Addition of TMZ to the cells resulted in a 36%, 48%, 60%, 64% and 60% induction of senescence (Fig 6A and 6B). These results correlate with an NSC666715-mediated increase in the accumulation of AP sites and increased p53/p21 activity with enhanced senescence in TMZ-treated HCT116 cells.

### TMZ-induced senescence is p53/p21 dependent

**Fig 7:**
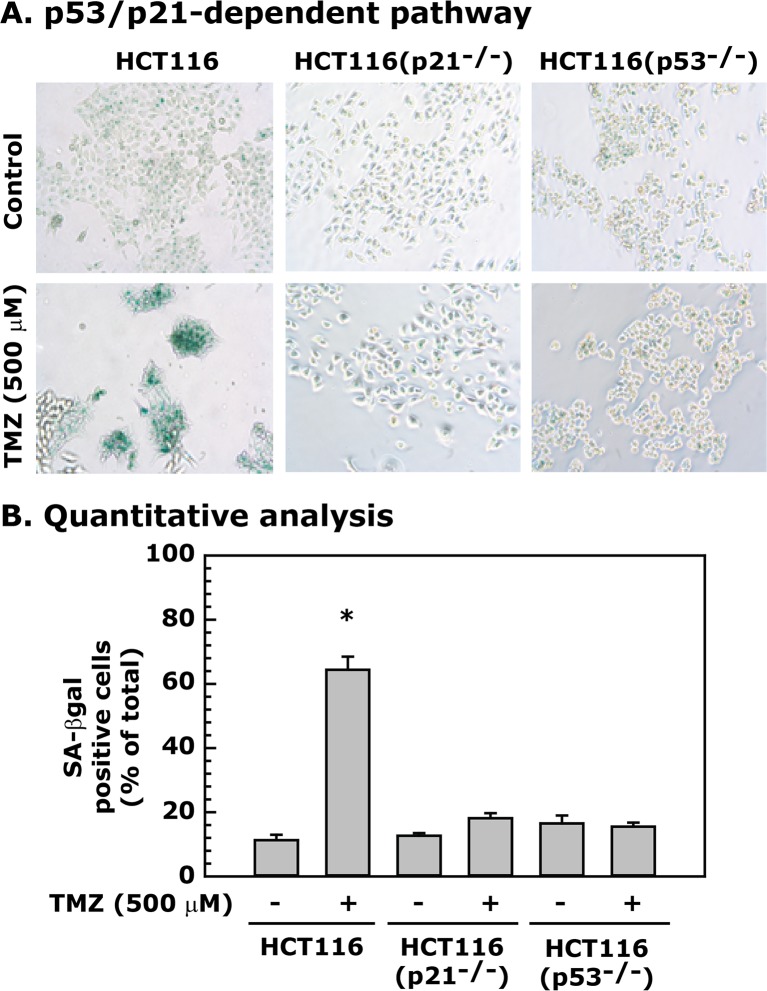
TMZ-induced senescence in HCT116 cells is dependent upon the p53/p21 pathway. HCT116 cells with or without p53 and p21 expression were treated with 500 μM TMZ for 48 h, and then processed for SA-βgal staining. **Panel A** shows the involvement of p53 and p21 in TMZ-induced senescence. **Panel B** represents quantitative analysis of the data, which are presented as mean ± SE of four different estimations. * = Significantly different than the untreated control (p<0.05).

After treatment of *p53* and *p21* gene knockout HCT116(p53^-/-^) and HCT116(p21^-/-^) cell lines [25, 40] with 500 μM of TMZ for 48 h, we observed a robust increase in the SA-βgal staining in HCT116 cells and markedly lower staining in both the HCT116(p53^-/-^) and HCT116(p21^-/-^) cell lines (Fig 7A and 7B). These results suggest that p53-dependent p21 activation is required for TMZ-induced senescence in HCT116 cells.

### PFTα blocks TMZ-induced senescence in HCT116 cells with or without NSC666715 treatment

**Fig 8:**
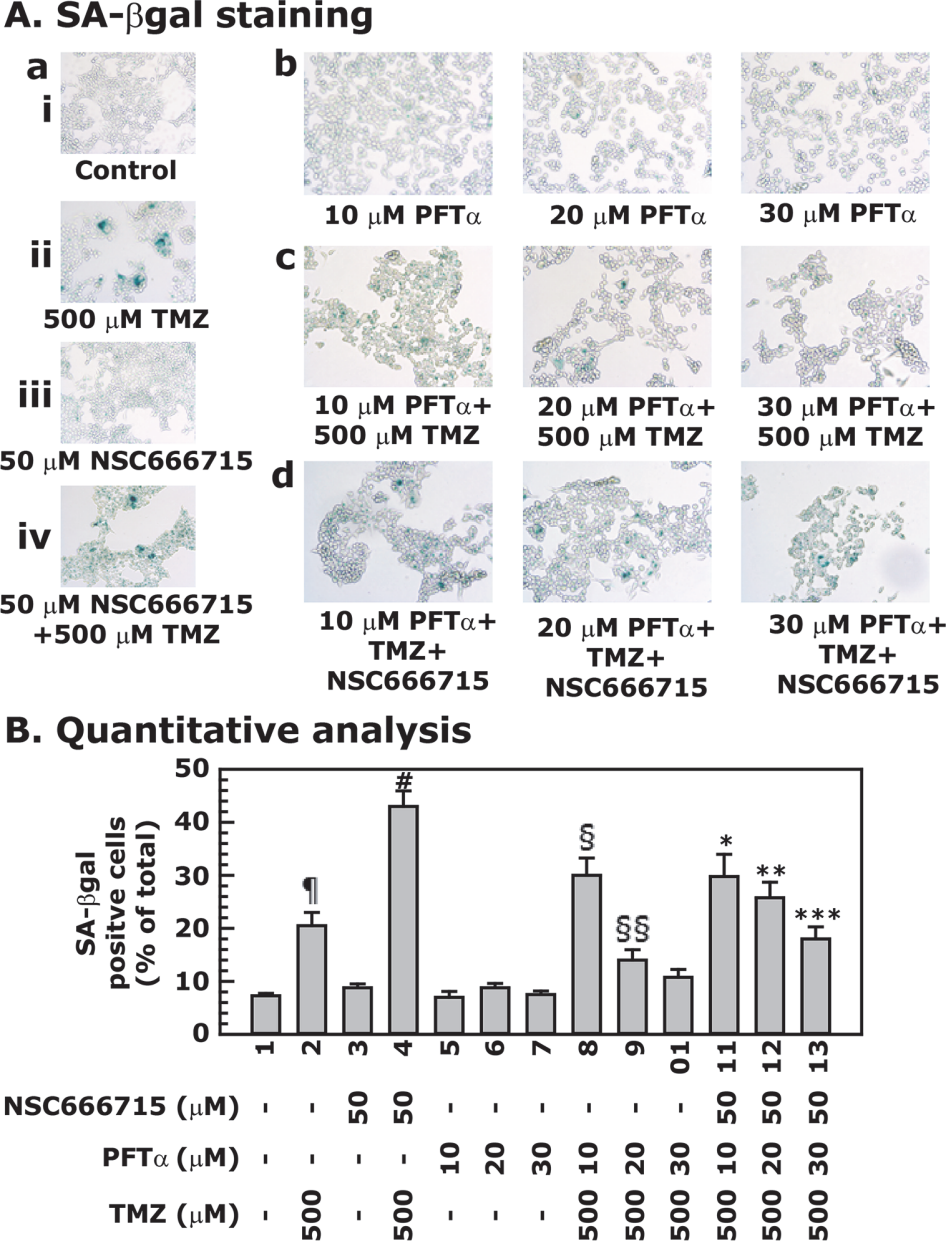
PFTα decreases TMZ and NSC666715-induced senescence in HCT116 cells. HCT116 cells were pretreated with different concentrations PFTα (10–30 μM) and/or 50 μM of NSC666715 for 2 h followed by the treatment with 500 μM TMZ for an additional 48 h. **Panel A** shows SA-βgal staining of the cells. **Panel B** represents the quantitative analysis of the number of SA-βgal positive cells. Data are presented as mean ± SE of four different estimations. ^¶^ = Significantly different than the untreated control (p<0.05). ^#^ = Significantly different than the 50 μM NSC666715 alone treated group (p<0.05). ^§^ and ^§§^ = Significantly different than the 10 and 20 μM PFTα alone treated groups, respectively, (p<0.05). *, ** and *** = Significantly different than the 500 μM TMZ in combination with 10, 20 and 30 μM PFTα treated groups, respectively, (p<0.05).

To further establish that the enhanced senescence in HCT116 after treatment with TMZ alone or in combination with NSC666715 requires the p53/p21 pathway, we examined SA-βgal staining in HCT116 cells treated with PFTα, NSC666715 and TMZ alone or in combinations (Fig 8A and 8B). The treatment conditions were the same as in the experiments above. Treatment with NSC666715 and PFTα alone did not show any increase in SA-βgal staining (Fig 8A[aiii] and 8A[b]) as compared to control (Fig 8A[ai]). PFTα treatment reduced TMZ-induced SA-βgal staining of HCT116 cells in a dose-dependent manner (compare Fig 8A[aii] with 8A[c]). Furthermore, the enhancement of SA-βgal staining after TMZ and NSC666715 combination treatment was also decreased when HCT116 cells were pretreated with different concentrations of PFTα (compare Fig 8A[aiv] with 8A[d]). These results further established the role of the p53/p21 pathway in TMZ-induced senescence in HCT116 cells, and this indicates that NSC666715-mediated BER signaling is dependent upon the p53/p21 pathway.

### TMZ-induced senescence sets the stage for apoptosis in HCT116 cells by inducing S-phase cell cycle arrest

**Fig 9:**
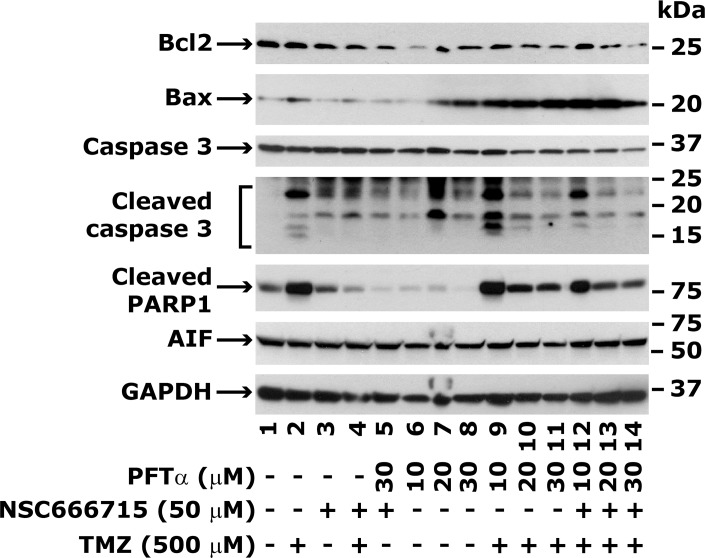
Effect of NSC666715 and PFTα on TMZ-induced levels of apoptosis-related proteins. The HCT116 cells were pretreated with different concentrations of PFTα and 50 μM NSC666715 for 2 h and then with 500 μM TMZ alone or in combination for an additional 48 h. Cells were harvested and the cellular lysates were prepared and processed for Western blot analysis. The Western blot analysis data are representative of two different experiments.

The cell cycle analyses suggest that HCT116 cells treated with TMZ are beginning to proceed from the G_2_/M phase into apoptosis at 48 h after treatment (Table 1). However, the results showed an unchanged level of the anti-apoptotic protein Bcl2 and an increased level of pro-apoptotic protein Bax (Fig 9, compare lane 1 with 2). NSC666715 treatment alone did not show any effect on Bcl2 or Bax levels, but in combination with TMZ, Bcl2 levels were decreased (Fig 9, compare lane 1 with 3 and 4). PFTα treatment in combination with NSC666715 or alone decreased Bcl2 levels and increased Bax levels in HCT116 cells (Fig 9, compare lane 1 with 5 and 6–8, respectively). The protein level of Bcl2 was decreased and Bax was increased once the TMZ treatment was combined with PFTα and NSC666715 (Fig 9, compare lane 1 with 9–11 and 12–14, respectively). These results indicate that TMZ- and NSC666715-treated cells are poised to enter apoptosis even in the presence of PFTα.

Alterations in Bcl2 and Bax levels upon TMZ-induced apoptosis also correlated with cleaved caspase 3 and PARP1 levels, and this suggests that TMZ-induced HCT116 cells are destined for apoptosis instead of prolonged survival. The expression level of apoptosis inducing factor (AIF), a mediator of caspase-independent apoptosis [41], in HCT116 cells after TMZ treatment with or without the combination of NSC666715 and PFTα treatment also showed that AIF levels were not significantly changed (Fig 9). These results suggest that the AIF-mediated pathway is not functional for TMZ-induced apoptosis in HCT116 cells.

## Discussion

TMZ is a Food and Drug Administration (FDA) approved drug for the treatment of glioblastoma [42]. A Phase II clinical study of TMZ in pre-selected advanced aerodigestive tract cancers has also been recently completed by Schering-Plough, Kenilworth, NJ, with a partial response outcome (http://clinicaltrials.gov/ct2/show/NCT00423150). In a separate Phase I clinical study of TMZ, the observed partial response of metastatic colorectal cancer to the drug was likely due to considerable tumor resistance [43]. To overcome TMZ resistance, another Phase II clinical study was performed in which lomeguatrib was combined with TMZ; however, the results were not statistically significant [44]. Thus, there is an urgent need for the development of a new strategy for enhancing the efficacy of TMZ. The mechanism of action of TMZ involves the production of strand breaks during BER-mediated repair of N^7^-MeG, N^3^-MeA and N^3^-MeG adducts, which are efficiently repaired. The interruption of the BER pathway can contribute to TMZ cytotoxicity due to the accumulation of AP sites. Unrepaired AP sites will then generate strand breaks that lead to cell death [18–21, 45].

Our proposed approach of combining SMI NSC666715 and/or its analogs with TMZ is novel because it can affect CRCs with both wild-type and mutant *APC* genes since the target of NSC666715 is the Pol-β. Our recent studies show that at low doses, NSC666715 can overcome TMZ-induced resistance and increase its efficacy against CRC [17]. We have described how NSC666715-mediated blockade of BER causes the accumulation of TMZ-induced AP sites, and that if these AP sites are not repaired, DSBs occur. The accumulated DSBs can then induce p53/p21 signaling resulting in S-G_2_/M phase cell cycle arrest and replicative senescence. In the glioma study, TMZ treatment activated three pathways in succession: autophagy, senescence and apoptosis [46].

Our study provides a pre-clinical approach for the development of new chemotherapeutic agents, which may facilitate the improvement of conventional colon cancer treatment. Our initial findings indicate that the strategy of combining NSC666715 with TMZ seems to effectively block the growth of both MMR-proficient and MMR-deficient colon cancer cells *in vitro* and *in vivo* (data not shown), as we have described in our previous studies [17]. This is noteworthy because MMR-deficient colorectal cancers pose a greater risk of resistance to DNA-alkylating drugs due to overexpression of MGMT or MMR-deficiency [47–49]. Cells deficient in MGMT are unable to process O^6^MeG during DNA synthesis [47]. The G:T mismatch is then repaired by the MMR pathway [48]. If O^6^MeG is not repaired before the re-synthesis step in MMR, it is believed that the repetitive cycle of futile MMR results in the generation of tertiary lesions, most likely gapped DNA. This then gives rise to DSBs in the DNA that elicit a cell death response [16, 49]. Thus, the blockade of repair of TMZ-induced N^7^-MeG, N^3^-MeA and N^3^-MeG lesions by NSC666715 causes much higher cytotoxicity than the mutagenic lesions of O^6^-MeG. The unrepaired N^7^-MeG, N^3^-MeA and N^3^-MeG lesions will accumulate and lead to single-strand DNA breaks (SSBs), stall the DNA replication fork and form DSBs during S phase. The persistent DSBs ultimately will trigger apoptosis [19].

The two types of cell senescence are replicative and accelerated [50–53]. Replicative senescence is a state of irreversible growth arrest of cells after consecutive cell division that can be triggered by telomere shortening and involves the p53/p21 pathway. Replicative senescence encompasses the DNA damage response mechanism [52, 54] involving the ATM/ATR kinases that leads to the phosphorylation of Ser139 of histone γ-H2AX [55, 56]. This phosphorylation event is believed to facilitate the assembly of nuclear foci that contain numerous DNA repair factors, including phospho-γ-H2AX, 53BP1, MDC1, NBS1, and phospho-SMC1. These DNA damage-induced foci can persist for months after growth arrest [56]. The DNA damage-induced activation of Chk1/Chk2 also stabilizes p53, which in turn activates *p21(Waf-1/Cip1)* gene expression in cells undergoing replicative senescence. Inhibition of the activity of cyclin-dependent kinases by p21 blocks E2F-dependent transcription by preventing the phosphorylation of Rb. The latter cascade of events then leads to permanent cell cycle arrest.

In glioma cells, a cyclin-dependent kinase (Cdk) inhibitor, flavopiridol, has been shown to potentiate the cytotoxicity of TMZ in a p53-independent manner. It induces cell death by mitotic catastrophe and/or senescence-like growth arrest through the suppression of key proteins at the G_2_-M transition, accumulation of the cells exclusively at the G_2_ phase, and an increase in DSBs [57–59]. In earlier studies, we have observed a conversion of the p53/p21 pathway from senescence to apoptosis in HCT116 cells after treatment with N-methyl-N’-nitro-N-nitrosoguanine (MNNG) [34]. In previous studies, we found that treatment of HCT116 cells with higher concentrations of MNNG-induced senescence that was linked with the loss of telomeric DNA. The results suggested that the loss of telomeric DNA by two-fold favors G_2_/M arrest and apoptosis in a p53/p21-dependent manner [34, 60]. In the present study, we found that TMZ- and NSC666715-induced senescence is dependent upon the p53/p21 pathway in HCT116 cells. This was supported by the use of p53^-/-^ and p21^-/-^ HCT116 cell lines and by using PFTα, a pharmacologic inhibitor of p53 activity. However, studies have shown that after MNNG and TMZ treatment glioblastoma cells underwent multiple cell cycles, maintained their metabolic activity, and had a prolonged period prior to cell death that involved the accumulation of AIF within the nucleus [61]. However, in our studies with HCT116 cells, the AIF pathway does not seem to be active after treatment with TMZ alone or in combination with NSC666715 and PFTα.

These results provide a guide for the development of a target-defined strategy for chemotherapy that will be based on the mechanisms of action of NSC666715 and TMZ. Findings will also identify how these mechanisms are affected in the context of different molecular defects in APC, p53 and p21 related to the senescence, apoptosis, and the development of resistance. The mechanisms by which NSC666715 and TMZ cooperate to suppress cancer cell proliferation and viability are complex and multifaceted. Future studies will be directed toward determining which of these mechanisms is most important in suppressing tumor growth *in vivo*.
